# Unintentional Injuries Requiring Medical Attention in Low-Income and Middle-Income Countries: Evidence from Nationally Representative surveys in 12 Countries

**DOI:** 10.1007/s44197-025-00420-y

**Published:** 2025-06-10

**Authors:** Leila Ghalichi, Albertino Damasceno, David Flood, Pascal Geldsetzer, Mongal Gurung, Maja Marcus, Kibachio Joseph Mwangi, Sebastian Vollmer, Michaela Theilmann, Justine Davies

**Affiliations:** 1 Department of Applied Health Sciences, College of Medicine and Health, University of Birmingham, Birmingham, UK; 2 Faculty of Medicine, Eduardo Mondlane University, Maputo, Mozambique; 3 Department of Internal Medicine, University of Michigan, Ann Arbor, MI USA; 4 Division of Primary Care and Population Health, Stanford University, Stanford, CA USA; 5 Policy and Planning Division, Ministry of Health, Thimphu, Bhutan; 6 Division of Infectious Diseases, Brigham and Women’s Hospital, Harvard Medical School, Boston, MA USA; 7 Division of Non-Communicable Diseases, Ministry of Health, Nairobi, Kenya; 8 Department of Economics and Centre for Modern Indian Studies, University of Goettingen, Göttingen, Germany

**Keywords:** Injury, Global Health, Equity, LMIC

## Abstract

**Background:**

Despite a high burden of injuries in low-income and middle-income countries (LMICs), a lack of empirical evidence on mechanism, location, and distribution of unintentional injuries requiring medical attention (hereafter injuries) hinders informed health system policy development.

**Methods:**

Using individual-level data from nationally representative surveys conducted in LMICs between 2014–2019, we describe the weighted annual prevalence of non-fatal injuries, their mechanisms, environments in which they occur, and characteristics of people injured, in individuals aged 15–64 years. Multivariable logistic regression models were estimated to evaluate associations of injuries with individual-level characteristics.

**Results:**

We included data on 47,747 participants from 12 LMICs in four WHO regions. The weighted prevalence of non-fatal injuries in the past year was 6.8% (95% CI: 6.3%-7.2%); men suffered a greater prevalence of injuries than women (8.3% [95% CI 7.6%-9.0%] vs. 5.4% [95% CI 5.0%-5.9%], respectively). In the multivariable logistic regression, the odds of having any injury were lower among females and married people and higher among individuals with some primary education.

Prevalence of non-road traffic collision injuries was almost threefold that of road traffic collision (RTC) injuries: 5.6% (5.2%-6.0%) vs. 1.7% (1.5%-2.0%). When considering non-RTC injuries, falls were the most common mechanism (47.0%, 95% CI 44.0%-50.1%), and homes were the most common location (38.1%, 95% CI 34.9%-41.4%), followed by road (17.9%, 95% CI 15.7%-20.4%), and workplace (17.4%, 95% CI 15.2%-19.8%). The largest proportion (23.2%, [95% CI 20.6%-25.9%]) of non-RTC injuries happened to women at home.

**Conclusion:**

Non-RTC injuries, in particular falls, predominate in this population. This highlights a neglected side of injuries, many of which happen at home to women, whereas global attention tends to focus on RTCs. Data on all mechanisms of injuries and care-seeking behaviour after injuries are required for health system planning.

## Introduction

Injuries account for approximately 10% of the global burden of disease. [1] Yet, injuries and their sequelae remain neglected in policy and research agendas. [2] Among injuries, road traffic collisions (RTC) receive a substantial amount of global attention, [3] as exemplified by the Sustainable Development Goal (SDG) target 3.6 to reduce the number of deaths and injuries from RTCs by 50% by 2030 [4] and the United Nations declaring a second global decade of Action for Road Safety (2021 to 2030). [5] Non-RTC related injuries, such as falls, cuts, or burns, receive comparatively less global attention than RTCs. However, the predominance of injuries as emergency conditions and the ratification of the 76 th World Health Assembly (WHA) resolution for integrated emergency, critical, and operative care within universal health coverage require the global community to focus on all injury mechanisms. To enable this, reliable data on the prevalence of injuries, where and how they occur, and which population groups are most at risk is crucial. [6, 7].

The lack of evidence on which population groups are predominantly affected by injuries and in which contexts injuries occur is particularly pronounced in low-income and middle-income countries (LMICs), which bear 85% of global injury deaths and disease burden. [6, 8] There exist some country-level prevalence estimates resulting from large-scale, population-level modelling. [9, 10] However, empirical evidence on injury prevalence patterns, disaggregated by population groups to identify those at high risk, is central to health system planning and policy development, including resource allocation. [11].

We used nationally representative survey data on adults aged 15–64 from 12 LMICs to describe the prevalence of non-fatal unintentional injuries requiring medical attention, which is indicative of the injuries being non-trivial. We explored injury patterns based on location of injury and mechanism, and estimated their association with country economic status and individual socio-demographic characteristics.

## Method

### Data Sources

Our analytical dataset contains individual-level data from the World Health Organization’s “STEPwise approach to NCD risk factor surveillance” (STEPS) [12] surveys conducted in LMICs between 2014 and 2019. Each survey employed a multistage cluster sampling resulting in a nationally representative sample of the sampled age range. [13] We used the harmonized dataset from the Global Health and Population Project on Access to Care for Cardiometabolic Disease (HPACC). [14] The data search strategy is described in detail elsewhere. [14] In brief, a systematic search identified all nationally representative surveys conducted in LMICs after 2005 with information on non-communicable diseases and their risk factors available. We restricted the dataset to surveys conducted in the 10 years before data were locked for analysis, and which contained data on RTC and non-RTC injuries requiring medical care. Although self-reported, the requirement for medical care indicates the non-triviality of injuries. Based on this procedure, our study included only surveys from the WHO STEPS collection conducted in LMICs between 2014 and 2024.

As part of the STEPS survey instrument injury module, participants were asked “In the past 12 months, have you been involved in a road traffic crash as a driver, passenger, pedestrian, or cyclist?” and if yes, “Did you have any injuries in this road traffic crash which required medical attention?”. The next question asked: “In the past 12 months, were you injured accidentally, other than the road traffic crashes, which required medical attention?”, followed up by questions asking about the mechanism (fall, cut, burn, animal bite, near-drowning, poisoning, and other) and location (home, school, workplace, road, farm, sport areas, and other) of the most serious non-RTC injury. We restricted the sample to individuals aged 15–64 years because this is the age range common to all surveys.

### Outcome Variables

Our main outcome was self-reported occurrence of an unintentional non-fatal injury requiring medical attention (hereafter termed “injuries”) in the past 12 months. Respondents were considered as having an injury if they answered positively to one or both of the two aforementioned questions. We compare RTC and non-RTC injuries as well as the following non-RTC injury mechanisms: fall, cut, burn, animal bite, near-drowning, and poisoning. For non-RTC injuries, we analyse the location where they occurred: home, school, workplace, road, farm, sports areas, or other locations.

### Exposure Variables

We included the following exposure variables: age, sex, marital status, education, and household residency. Age was included as ten-year categories (15–24, 25–34, 35–44, 45–54, 55–64). Marital status was categorised as single (never married, divorced, or widowed) and married (married or cohabiting). Education was categorised into three levels: no formal education, some primary education or primary school completed [henceforth: primary education], or some high school or above. Household residency was either rural or urban. Countries were grouped based on World Bank income group classification at the time of the survey, as low-income, lower-middle-income, or upper-middle-income.[15].

### Statistical Analysis

First, we estimated the weighted injury prevalence in the pooled sample, by individual-level characteristics, and by World Bank income group and described the distribution of injury mechanisms and locations of injuries by sex and age groups. Second, we fit multivariable weighted logistic regression models with robust standard errors clustered at the primary sampling unit to estimate associations between outcomes and exposures. In the analyses pooling multiple countries, we weighted each country according to its population size in 2015. We accounted for the complex multi-stage sampling designs using the svyset command in Stata 18. In each analysis, we included only complete cases.

## Results

### Sample Characteristics

Our sample consisted of 47,747 participants from 12 LMICs across four different WHO regions (Europe, South-East Asia, Africa, and Eastern Mediterranean). The countries included were Azerbaijan, Bhutan, Eswatini, Ethiopia, Guyana, Kenya, Mozambique, Nepal, São Tomé and Príncipe, Timor-Leste, Turkmenistan, and Ukraine (Table 1). Data on rural/urban residence was not available for Eswatini and Timor-Leste; for this reason, we did not include this variable in the main analysis but present the results in the appendix. Ethiopia did not have information on injury location. All variables had less than 10% of missing values.

**Table 1:** Sample size, data collection tools and time span of the countries

	World bank income category	WHO region	Number of participants (15–64)	Data available on residence	Survey year
Azerbaijan	Upper middle	EURO	2,608	Yes	2017
Bhutan	Lower middle	SEARO	5,314	Yes	2019
Eswatini	Lower middle	AFRO	3,350	No	2014
Ethiopia	Low	AFRO	9,479	Yes	2015
Guyana	Upper middle	AMRO	2,509	Yes	2016
Kenya	Lower middle	AFRO	4,282	Yes	2015
Mozambique	Low	AFRO	2,590	Yes	2014–2015
Nepal	Lower middle	SEARO	5,281	Yes	2019
São Tomé and Príncipe	Lower middle	AFRO	2,344	Yes	2019
Timor-Leste	Lower middle	SEARO	2,407	No	2014
Turkmenistan	Upper middle	EURO	3,907	Yes	2018
Ukraine	Lower middle	EURO	3,676	Yes	2019

All countries use the WHO STEPwise (STEPS) approach to surveillance.

EURO: WHO Regional Office for the Europe

SEARO: WHO Regional Office for the South-East Asia

AFRO: WHO Regional Office for the Africa

EMRO: WHO Regional Office for the Eastern Mediterranean

### Prevalence Estimates

Of the 47,747 individuals in our sample, 46,800 (98.0%) had data on injuries. Among these, 52.6% were female, and the average age was 35.2 years (SD: 12.97). 34.8% were single, 50.7% had high school education or above, 59.1% resided in rural area, and 58.3% lived in a lower-middle income country (Table 2). Among the 2,761 respondents who reported any injury, 42.2% were female and the average age was 34.6 years (SD: 12.76), 42.2% were single, and 49.3% % had high school education or above, 54.2% resided in rural area and 64.0% in a lower-middle income country (Table 2).

**Table 2:** Characteristics of participants from all included countries

	All survey participants Pooled sample	Participants with injuries (%)	Injury prevalence (95% CI)
	N = 46,800	N = 2761	6.8% (6.3%−7.2%)
WB country income group [N, (%)]			
LIC	11,916 (16.7%)	559 (19.5%)	7.9% (6.8%−9.2%)
L-MIC	25,947 (58.3%)	1901 (64.0%)	7.4% (6.8%−8.1%)
U-MIC	8,937 (25.0%)	301 (16.5%)	4.5% (3.8%−5.2%)
Age			
15–24	8,487 (25.3%)	541 (27.4%)	7.4% (6.5%−8.3%)
25–34	12,698 (28.1%)	727 (27.1%)	6.5% (5.9%−7.2%)
35–44	10,855 (21.0%)	628 (21.0%)	6.8% (6.1%−7.7%)
45–54	8,243 (14.7%)	515 (15.8%)	7.2% (6.1%−8.6%)
55–64	6,517 (10.9%)	350 (8.7%)	5.4% (4.7%−6.2%)
Sex [N, (%)]			
Male	18,370 (47.4%)	1,415 (57.8%)	8.3% (7.6%−9.0%)
Female	28,430 (52.6%)	1,346 (42.2%)	5.4% (5.0%−5.9%)
Marital Status [N, (%)]			
Single	14,757 (34.8%)	1,046 (42.2%)	7.8% (7.1%−8.5%)
Not single	31,947 (65.2%)	1,710 (57.8%)	6.2% (5.7%−6.7%)
Education [N, (%)]			
No formal education	11,373 (16.5%)	466 (10.9%)	4.9% (4.3%−5.7%)
Primary education	14,576 (32.8%)	1,053 (39.8%)	8.1% (7.3%−8.9%)
Some High School or above	20,798 (50.7%)	1,242 (49.3%)	6.5% (5.9%−7.1%)
Residence [N, (%)] (available for 10 countries)			
Urban	16,347 (40.9%)	1,144 (45.8%)	7.7% (6.8%−8.6%)
Rural	25,027 (59.1%)	1,298 (54.2%)	6.3% (5.8%−6.9%)

Estimates are weighted. Abbreviations: LIC = low-income country, L-MIC = lower-middle income country, U-MIC = upper-middle income country.

The weighted prevalence of injuries in the past year was 6.8% (95% CI 6.3%−7.2%). The injury prevalence ranged from 4.5% in upper-middle income countries to 7.9% in low-income countries. It was higher among males than females (8.3% [95% CI 7.6%−9.0%] vs. 5.4% [95% CI 5.0%−5.9%], respectively) and highest in the 15–24 years age group and lowest in 55–64 years age group. The prevalence was also higher among singles, individuals with primary education, and those living in urban areas (Table 2). Disaggregating prevalence estimates by age and sex showed that the prevalence was higher among males than females across all age groups except those aged 55–64 years (Appendix 1).

The prevalence of RTC-related injuries was 1.7% (95% CI 1.5%−2.0%) and that of non-RTC injuries was 5.6% (95% CI 5.2%−6.0%; p-value for difference: < 0.001). Both RTC and non-RTC injuries were more prevalent among males than among females (2.4% (95% CI 2.0%−2.9%) vs. 1.1% (95% CI 1.0%−1.3%) for RTC and 6.6% (95% CI 6.0%−7.2%) vs. 4.7% (95% CI 4.3%−5.2%) for non-RTC injuries) (Table 3). Amongst those injured, the proportion of females was higher among those experiencing non-RTC (44.2%, 95% CI 41.2%−47.3%) compared to RTC injuries (34.6%, 95% CI 29.2%−40.4%, p < 0.001).

**Table 3:** Weighted prevalence of injury mechanism in males and females, and mean age for each mechanism

Cause of Injury	Number	Weighted Prevalence	Weighted prevalence in Males	Weighted prevalence in Females	P value	Mean age of injured participants (SD)
Any Injury	2761	6.8% (6.3%−7.2%)	8.3% (7.6%−9.0%)	5.4%, (5.0%−5.9%)	< 0.001	34.6 (12.76)
RTC	682	1.7% (1.5%−2.0%)	2.4% (2.0%−2.9%)	1.1% (1.0%−1.3%)	< 0.001	35.6 (12.13)
Non-RTC*	2290	5.6% (5.2%−6.0%)	6.6% (6.0%−7.2%)	4.7% (4.3%−5.2%)	< 0.001	34.2 (12.84)
Fall	1067	2.6% (2.3%−2.8%)	3.0% (2.6%−3.4%)	2.2% (1.9%−2.5%)	< 0.001	34.5 (13.10)
Cut	669	1.7% (1.5%−2.0%)	1.9% (1.6%−2.3%)	1.5% (1.3%−1.9%)	0.065	33.6 (12.62)
Burn	121	0.3% (0.2%−0.4%)	0.2% (0.2%−0.4%)	0.3% (0.2%−0.4%)	0.340	32.4 (12.46)
Animal Bite	111	0.3% (0.2%−0.3%)	0.2% (0.1%−0.3%)	0.3% (0.2%−0.4%)	0.283	36.8 (13.13)
Near-drowning	45	0.1% (0.1%−0.2%)	0.2% (0.1%−0.3%)	0.0% (0.0%−0.1%)	< 0.001	36.2 (13.01)
Poisoning	30	0.0% (0.0%−0.1%)	0.1% (0.1%−0.2%)	0.0% (0.0%−0.1%)	0.029	36.9 (9.40)
Other	201	0.5% (0.4%−0.5%)	0.7% (0.5%−0.9%)	0.2% (0.2%−0.3%)	< 0.001	34.4 (12.61)

95% confidence intervals are displayed in brackets.

* Mechanism of non-RTC was missing in some cases, thus the sum of mechanisms is less than total non-RTCs.

** The p-value reflects the statistical significance of the difference of each injury mechanism between male and females.

Falls were the most common injury mechanism with a prevalence of 2.6% (95% CI 2.3%−2.8%), followed by RTC (1.7% [95% CI 1.5%−2.0%]), and cuts (1.7% [95% CI 1.5%−2.0%]) (Table 3). Other causes, including burns, animal bites, drowning, poisoning, and other mechanisms, had a combined prevalence of 1.2%. Except for burns and animal bites, the prevalences of all injury mechanisms were higher among males than females. The mean age was similar across different injury mechanisms (Appendix 3).

**Fig. 1:**
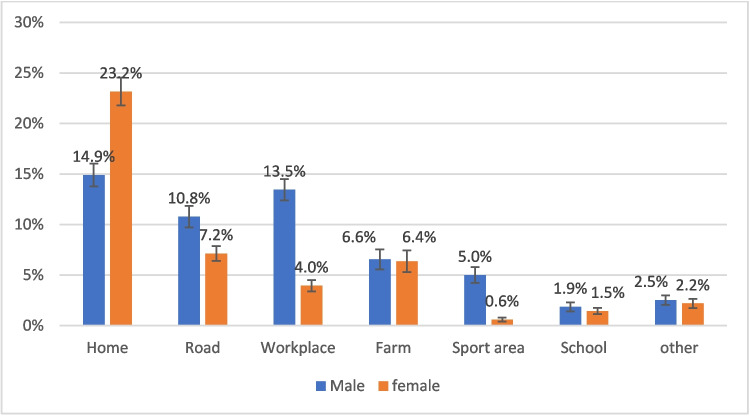
Distribution of non-RTC injury locations for male and female participants

In the 11 countries with information on injury location, 38.1% (95% CI 34.9%−41.4%) of non-RTC injuries occurred at home, followed by road (17.9%, 95% CI 15.7%−20.4%), and workplace (17.4%, 95% CI 15.2%−19.8%) (Appendix 4). Among women, the majority of injuries occurred at home (51.6%, 95% CI 46.6%−56.6%) whereas among men, both home (27.1%, 95% CI 23.4%−31.0%) and workplace (24.4%, 95%CI 21%−28.1%) were the most common locations. When considering all individuals injured in non-RTC incidents, the largest proportion (23.2%, [95% CI 20.6%−25.9%)] happened to women at home (Fig. 1). Appendix 5 shows the distribution of non-RTC injury location by age groups.

**Fig. 2:**
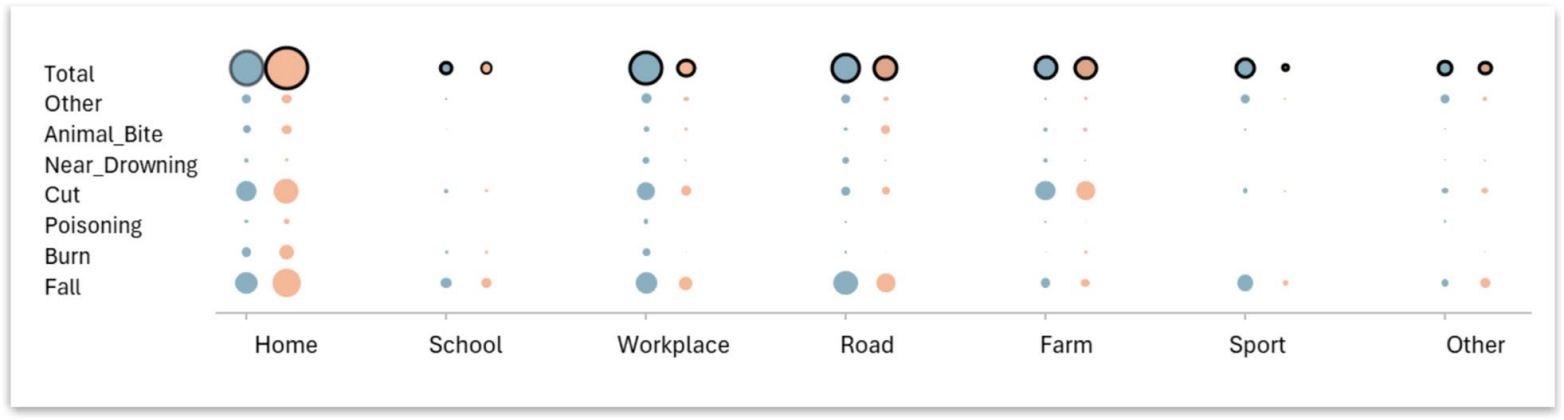
Mechanisms and locations of non-RTC injuries in males and females. The size of the circles represents the prevalence in males (blue) and females (pink) respectively

When considering locations and mechanisms of non-RTC injuries, falls at home were the most common, accounting for 16.6% (95% CI 14.4%−19.1%), followed by cuts at home (13.0%, 95% CI 10.9%−15.4%), and falls on the road (12.3%, 95% CI 10.4%−14.6%) (Fig. 2, Appendix 6). Among females, falls at home account for 22.7% (95% CI 19.1%−26.8%) of non-RTC injuries. Among males, the most common non-RTC injury mechanism and location were falls on the road (13.6%, 95% CI 10.7%−17.1%) (Appendix 7).

The prevalence of total injuries, the proportions of RTC and non-RTC injuries, and distribution of injury types varied substantially between countries (Appendix 8).

### Regression Estimates

In the multivariable regression model, being female and married were associated with lower odds of having experienced an injury while having some primary education (compared to having no education) and being 35 to 54 years old (compared to the 55–64 age group) were associated with higher odds of injury (Table 4). When adding household residence to the model, living in a rural area was associated with lower odds of injury compared to urban areas in the 10 countries with data on residence available. Other point estimates and confidence intervals were comparable to those of the main analysis (Table 4). Looking at RCT and non-RCT injuries separately yields similar results (Appendix 2).

**Table 4:** Multivariable analysis of the associations of having any injury in the previous 12 months

	In 12 Countries (without residence variable)			In 10 Countries (with residence variable)		
	OR	95% CI	P value	OR	95% CI	P value
Age (Ref: 55–64)						
15–24	1.19	0.97–1.45	0.086	1.18	0.95–1.47	0.124
25–34	1.19	0.99–1.43	0.065	1.16	0.95–1.42	0.134
35–44	1.25	1.03–1.52	0.023	1.19	0.98–1.44	0.077
45–54	1.37	1.10–1.71	0.006	1.36	1.07–1.74	0.014
Sex (Ref: Male)						
Female	0.64	0.57–0.73	< 0.001	0.65	0.57–0.75	< 0.001
Marital Status (Ref: Single)						
Non-single	0.80	0.73–0.93	0.001	0.83	0.72–0.95	0.007
Education (Ref: no formal education)						
Primary education	1.52	1.40–2.03	< 0.001	1.57	1.30–1.90	< 0.001
Some High School or above	1.19	1.22–1.88	0.075	1.10	0.90–1.33	0.362
Rural (Ref: urban)				0.79	0.67–0.93	0.005

## Discussion

Using nationally representative data from 12 LMICs, we find that the prevalence of any kind of unintentional injury requiring medical attention in the past 12 months was 6.8%. The prevalence of non-RTC injuries requiring medical attention was more than three times that of RTC injuries, and the prevalence of falls alone was higher than RTCs. This finding is particularly notable because our study is limited to people between 15 and 64 years and does not cover children and elderly populations known to have a high incidence of fall-related injuries, [16, 17] and who commonly are the main target of fall prevention programs. [18, 19] Pedestrian or road-related falls are another prevalent category in our study. Despite being an actionable target in road safety policies, these falls are a neglected issue both in road safety research and in injury prevention strategies.[20, 21] Inadequate public space safety measures resulting in fall-related trauma on the road hinders outdoor physical activity, resulting in further adverse psychosocial and health outcomes. [17] Given the higher risk of falls among older adults and the increased severity of outcomes in this population, combined with the rapid ageing trends observed in LMICs, and the need for policies to increase activities as people age to reduce frailty and sarcopenia, [22, 23] falls represent a critically important issue for both research and policy development in these settings.

Given that falls impose a high economic burden on individuals, their families, and health systems, our evidence shows that falls should receive increased attention. [8, 24, 25] Interventions generally target children and elderly populations or address falls occurring in the workplace. [26] However, our study suggests that other age groups are at high risk, too, and more attention should be given to falls occurring elsewhere. Together, the evidence underscores the need for developing more effective policies to reduce fall risk tailored to individuals and locations through health education, improving home and environment safety, and investing in fall-related research. [27] Animal bites, burns, near-drowning, and other less common causes have all been the focus of research and policy development aimed at reducing their burden. [26, 28] However, the extent of the attention given to these injury mechanisms has not matched the scale of these global health challenges.

While global efforts for making roads a safer space are making progress, especially for moving vehicles, [29] we have found that homes are the most common place for non-RTC injuries to occur and our findings indicate the importance of programs aiming at safer home environments. We have shown that within the home, injuries are most often suffered by women and most often due to falls, cuts, or burns. This might highlight a neglected aspect of injuries underrepresented in medical records and self-report surveys and, as a result, not addressed in proportion to their severity and prevalence. In many LMICs, women may experience more delays and barriers in accessing health care, which might in turn affect health outcomes. [30–32] The STEPS module used in this study specifically refers to unintentional injuries and does not ask about domestic violence. Thus, despite finding a strikingly high prevalence of injuries among women, our findings likely underestimate the overall number of injuries affecting women in homes. The high prevalence of fall-related injuries at home among women calls for investigating the underlying causes before effective solutions are proposed. In general, home injuries are caused by the interaction of behavioural, physical, environmental, and social factors, and preventing them requires a systematic and holistic approach. [33] Many interventions, especially to prevent falls, are basic and easy to implement, such as fencing off or restricting access to dangerous areas and enforcement of building standards, but might not address all risks inherent to day to day life activities. [27].

Although injuries are known to be more prevalent among males,[9] we found the difference between sexes to be less prominent for non-RTC than RTCs. Observed sex disparities in injury occurrence are mainly attributed to risk-taking behaviours and occupational hazards, which are more prominent in men. Despite a higher injury prevalence among males, other studies have shown long-term health outcomes in women may be worse, with a lower quality of life and higher psychological morbidity after trauma for women compared with men. [34, 35] Some studies have even proposed developing separate injury severity scores for females and males due to underlying gender differences in risk factors, access to care, severity, and inflammatory responses. [36–38].

We found that injuries were numerically less prevalent in upper-middle-income countries than in low- and lower-middle-income countries. Despite the limited number of countries in each group, our result is in accordance with findings from other studies showing a global decline in injury burden with improved country socioeconomic index, although the pattern varies across injuries. [1] A previous study has demonstrated a decreasing trend of non-fatal RTCs from upper-middle-income to low-income-countries, accompanied by a decreased proportion of cyclists and increased proportion of drivers involved in RTCs. [11] The change in the composition of road users may partially explain the higher rate of non-fatal RTCs in low-income countries, despite the lower number of motor vehicles. [39] We also observed that having some or completed primary education was associated with a higher risk of injuries compared to no formal education. As education is the only measure of economic status in our study, the lower rate of injuries in individuals without formal education may be attributed to less exposure to injury risk factors such as vehicle ownership. Furthermore, individuals with no formal education may have lower exposure to certain injury risks resulting from specific types of occupation and social activities, as important contributors to inequality in injuries. [40].

Our findings highlight that injuries are not homogenous conditions, and that high-quality and granular empirical data can show variations in the locations, mechanisms, and types of injuries by the socio-demographic factors of the individuals who are injured. For the development and implementation of effective policies to prevent and develop health care systems for injuries, it is important that more granular data are widely available.

### Limitations

Our study has several limitations. Although the reported injuries were severe enough to require medical attention according to the respondent, we were not able to distinguish between minor and major injuries. The self-report nature of the surveys and the lack of medical confirmation of the requirement for medical attention are other limitations of the study. However, the fact that respondents sought care reflects the need for health systems to cover the health needs of these individuals. Our data only captures injuries requiring medical attention, but not those resulting in death. Thus, our prevalence estimates should be considered lower bounds as they only capture a subset of injuries. [41] Our study population excludes children and older adults due to the age ranges sampled for the STEP surveys. Education was the only socioeconomic measure available in this analysis.

## Conclusion

Non-fatal injuries are prevalent in the adults aged 15–64 in 12 LMICs. Among them, non-RTC injuries, in particular falls, predominate. This highlights a neglected aspect of injuries, many of which happen at home to women, and underscores the global community to prioritise prevention efforts, expansion of health coverage, and research activities.

## Figures and Tables

**Fig. 3:**
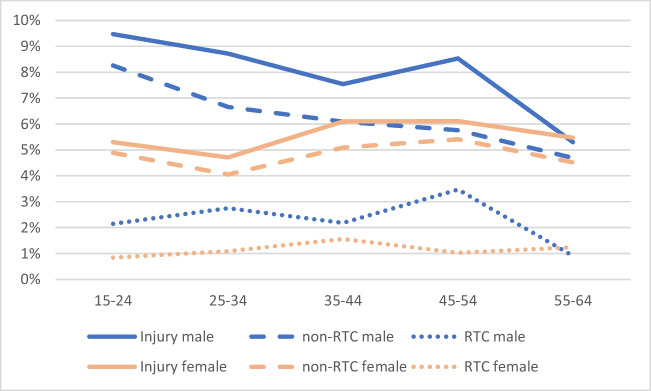
Prevalence of all injuries, RTCs and non-RTCs in different age groups in males and females. The prevalence of injuries was statistically different between males and females in all age groups except 55–64 years. For RTCs the difference was significant in 15–24 years, 25–34 years and 45–54., and for non-RTCs in those 15–24 years and 25–34 years

**Fig. 4:**
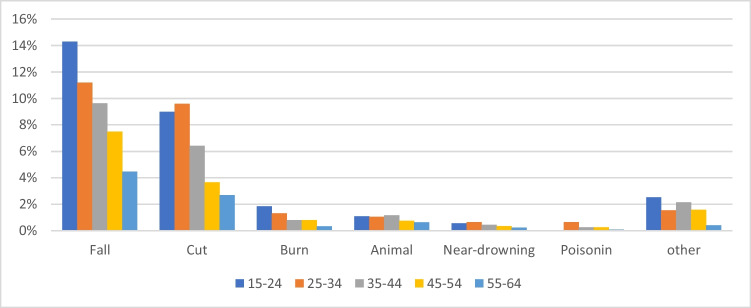
Distribution of non-RTC injuries based on age and mechanism of injury

**Fig. 5:**
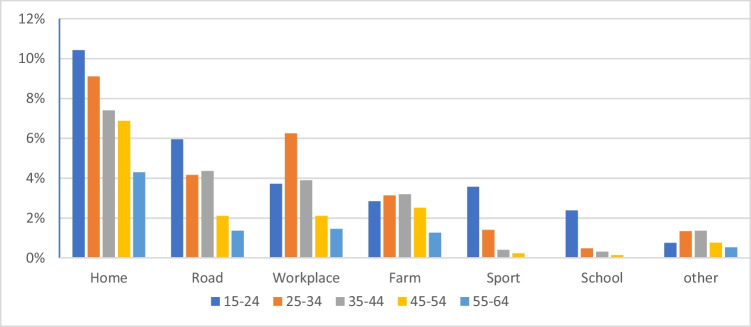
Distribution of non-RTC injuries based on age and location

**Table 9:**
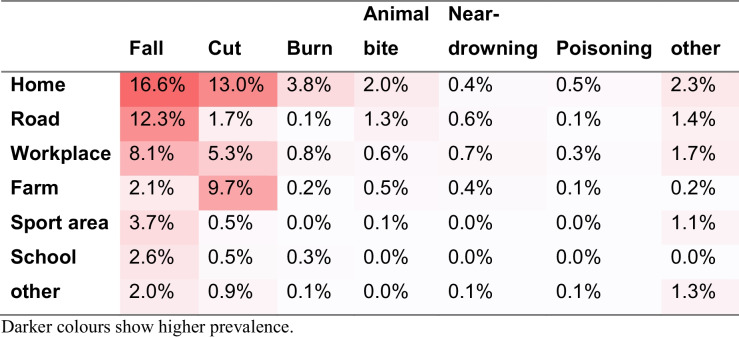
Combined distribution of injury type and location in respondents reporting having a non-RTC injury

**Fig. 6:**
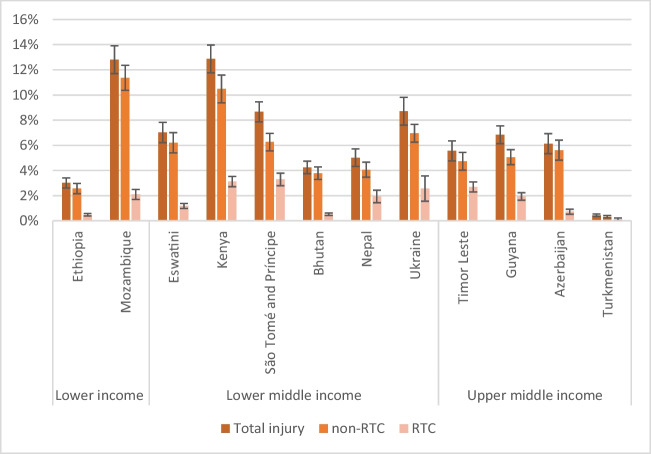
Prevalence of total injuries, RTCs and non-RTCs in 12 countries in three different World Bank Categories

**Fig. 7:**
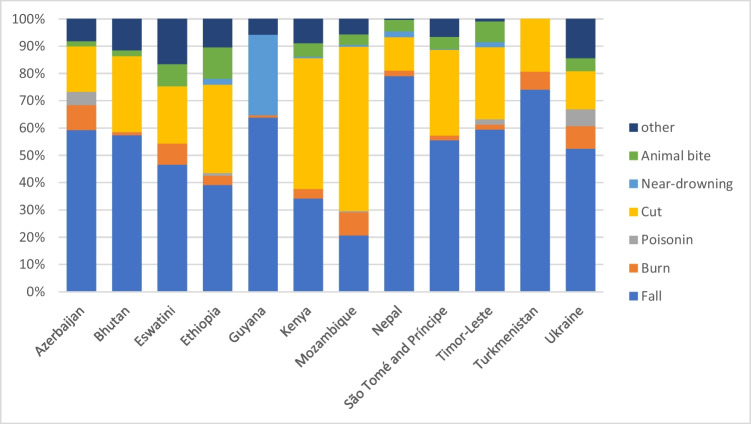
Prevalence of non-RTC injuries mechanisms in countries

## Data Availability

This study includes individual-level data from 12 countries. All this data is publicly available from the WHO NCD microdata repository (extranet.who.int/ncdsmicrodata). The HPACC harmonized dataset can be requested through https://dataverse.harvard.edu/dataverse/hpacc.

## Appendix 1. Prevalence of injuries by age groups in males and females.

Figure 3

Table 5

**Table 5:** Prevalence of injuries in different age groups in male and female

	Injury male	Injury female	P value	RTC male	RTC female	P value	non-RTC male	non-RTC female	P value
15–24	9.5%	5.3%	< 0.001	2.2%	0.8%	< 0.001	8.3%	4.9%	< 0.001
25–34	8.7%	4.7%	< 0.001	2.8%	1.1%	< 0.001	6.7%	4.1%	< 0.001
35–44	7.5%	6.1%	0.042	2.2%	1.6%	0.098	6.1%	5.1%	0.119
45–54	8.5%	6.1%	0.037	3.5%	1.0%	< 0.001	5.8%	5.4%	0.623
55–64	5.3%	5.5%	0.817	0.9%	1.2%	31.5%	4.7%	4.5%	0.814

## Appendix 2 Multivariable analysis of RTCs and non-RTCs

Table 6

**Table 6:** Multivariable analysis of the associations of having an RTC-related injury in the previous 12 months

	In 12 Countries (without residence variable)			In 10 Countries (with residence variable)		
	OR	95% CI	P value	OR	95% CI	P value
Age (Ref: 55–64)						
15–24	1.13	0.73–1.75	0.586	1.20	0.95–1.47	0.465
25–34	1.63	1.11–2.38	0.012	1.69	0.95–1.42	0.016
35–44	1.66	1.15–2.39	0.007	1.63	0.98–1.44	0.021
45–54	2.02	1.14–3.58	0.015	2.11	1.07–1.74	0.032
Sex (Ref: Male) Female	0.48	0.38–0.61	< 0.001	0.46	0.57–0.75	< 0.001
Marital status (Ref: Single) Non- Single	0.82	0.63–1.07	0.139	0.81	0.72–0.95	0.158
Education (Ref: no formal education)						
Primary education	1.97	1.34–2.88	< 0.001	2.35	1.30–1.90	< 0.001
Some High School or above	1.50	0.97–2.31	0.066	1.45	0.90–1.33	0.126
Residence (Ref: urban)				0.60	0.67–0.93	0.002

Table 7

**Table 7:** Multivariable analysis of the associations of having a non-RTC injury in the previous 12 months

	In 12 Countries (without residence variable)			In 10 Countries (with residence variable)		
	OR	95% CI	P value	OR	95% CI	P value
Age (Ref: 55–64)						
15–24	1.27	1.03–1.57	0.024	1.25	0.95–1.47	0.059
25–34	1.12	0.92–1.38	0.252	1.10	0.95–1.42	0.370
35–44	1.21	0.98–1.50	0.082	1.14	0.98–1.44	0.213
45–54	1.22	1.01–1.49	0.040	1.20	1.07–1.74	0.094
Sex (Ref: Male) Female	0.70	0.62–0.80	< 0.001	0.73	0.57–0.75	< 0.001
Marital status (Ref: Single) Non-single	0.81	0.70–0.92	0.002	0.82	0.72–0.95	0.006
Education (Ref: no formal education)						
Primary education	1.35	1.11–1.64	0.002	1.40	1.30–1.90	0.001
Some High School or above	1.07	0.88–1.29	0.496	1.03	0.90–1.33	0.787
Residence (Ref: urban)				0.86	0.67–0.93	0.089

## Appendix 3

Figure 4

## Appendix 4

Table 8

**Table 8:** Distribution of injuries based on location and sex

	total		Male		Female	
	Percent	95% CI	Percent	95% CI	Percent	95% CI
Home	38.1%	34.9%-41.4%	27.1%	23.4%-31.0%	51.6%	46.6%-56.6%
Road	17.9%	15.7%-20.4%	19.6%	16.2%-23.4%	15.9%	13.2%-19.1%
Workplace	17.4%	15.2%-19.8%	24.4%	21%-28.1%	8.8%	6.7%-11.5%
Farm	13.0%	10.5%-15.9%	11.9%	8.9%-15.7%	14.2%	10.4%-19.1%
Sport area	5.6%	4,2%-7.4%	9.1%	6.7%-12.1%	1.3%	0.7%-2.5%
School	3.3%	2.4%-4.5%	3.4%	2.1%-5.3%	3.2%	2.1%-4.9%
other	4.7%	3.6%-6.2%	4.6%	3.2%-6.5%	4.9%	3.2%-7.3%

## Appendix 5

Figure 5

## Appendix 6

Table 9

## Appendix 7

Table 10

**Table 10:** Combined distribution of injury type and location in respondents reporting having a non-RTC injury in A. females B. males

	Total	Fall	Cut	Burn	Animal bite	Near drowning	Poisoning	other
Home	51.6%	22.7%	17.0%	5.8%	2.5%	0.2%	0.8%	2.5%
Road	15.5%	10.7%	1.5%	0.0%	2.4%	0.2%	0.0%	0.7%
Workplace	8.9%	5.1%	2.6%	0.1%	0.3%	0.1%	0.0%	0.7%
Farm	14.7%	2.2%	10.8%	0.4%	0.7%	0.2%	0.1%	0.3%
Sport area	1.4%	1.1%	0.2%	0.0%	0.0%	0.0%	0.0%	0.1%
School	3.3%	2.6%	0.4%	0.3%	0.0%	0.0%	0.0%	0.0%
other	4.9%	3.0%	1.1%	0.1%	0.0%	0.2%	0.0%	0.5%
Home	27.8%	11.5%	9.6%	2.1%	1.5%	0.6%	0.3%	2.2%
Road	19.0%	13.6%	1.8%	0.2%	0.3%	1.0%	0.1%	2.0%
Workplace	24.6%	10.5%	7.6%	1.4%	0.8%	1.2%	0.6%	2.5%
Farm	3.4%	2.6%	0.5%	0.2%	0.0%	0.0%	0.0%	0.1%
Sport area	12.1%	2.1%	8.9%	0.0%	0.4%	0.5%	0.1%	0.1%
School	8.6%	5.9%	0.7%	0.0%	0.1%	0.0%	0.0%	1.9%
other	4.3%	1.2%	0.8%	0.0%	0.0%	0.1%	0.2%	2.0%

## Appendix 8. Prevalence of injury types in Countries.

Figure 6

Figure 7
